# Therapeutic Drug Monitoring for Lacosamide in Chinese Pediatric Patients with Epilepsy: Focus on Clinical Effectiveness, Tolerability and Drug Interactions

**DOI:** 10.7150/ijms.107660

**Published:** 2025-02-11

**Authors:** Fengqian Mao, Shunan Chen, Yani Hu, Suhong Wang, Meng Chen, Junjun Xu, Mingdong Yang, Jie Chen, Xiuping Zhu, Wei Hu, Feng Li, Lingyan Yu, Haibin Dai

**Affiliations:** 1 School of Pharmaceutical Sciences, Wenzhou Medical University, Wenzhou 325035, China.; 2 Department of Pharmacy, Second Affiliated Hospital, Zhejiang University School of Medicine, Hangzhou 310009, China.; 3 Department of Pediatrics, Second Affiliated Hospital, Zhejiang University School of Medicine, Hangzhou 310009, China.; 4 Research Center for Clinical Pharmacy, Zhejiang University, Hangzhou 310058, China.

**Keywords:** epilepsy, pediatric, lacosamide, plasma concentration range, therapeutic drug monitoring

## Abstract

**Objective:** To investigate the effectiveness and tolerability of lacosamide (LCM) and to select a better reference range for its concentration in plasma for Chinese pediatric patients with epilepsy. In addition, it is necessary to evaluate the potential determinates of LCM concentration.

**Methods:** Pediatric epilepsy patients using LCM were retrospective included. The clinical data of these patients were retrospectively reviewed, and the effectiveness at 3, 6, and 12 months after treatment was assessed. Drug concentrations from routine therapeutic drug monitoring (TDM) were also obtained. The trough concentration-to-dose ratio (C_0_/dose ratio) of LCM was compared among patients with various potential influencing factors. In addition, a new reference range was established based on the range in which the majority of patients were located and the proportion of responders within this range.

**Results:** A total of 153 pediatric epilepsy patients were finally included. The frequency of seizures decreased by ≥50% was 74.7%, 73.0%, and 71.2% at 3, 6, and 12 months, respectively. Adverse events (AEs) occurred in 53 patients, and most AEs were mild and moderate. The TDM data showed that it is reasonable to recommend using 2.5 to 6.5 µg/mL as the reference range. The C_0_/dose ratio was significantly associated with weight, but those aged 4 to 12 were significantly lower than those aged >12 years. In addition, LCM-antiepileptic drug (AED) interactions were observed. Oxcarbazepine and perampanel significantly decreased the C_0_/dose ratio of LCM.

**Conclusions**: LCM was efficacious in reducing seizure frequency and well tolerated in pediatric patients with epilepsy. The reference range 2.5-6.5 µg/mL, for routine LCM monitoring may be more applicable. As complex LCM-AED interactions were observed, it is necessary to monitor the plasma concentration.

## Introduction

Epilepsy is a common chronic neurological diseases affecting individuals globally.^1^ Approximately 2% of the population is affected by epilepsy (lifetime prevalence), and in the majority (three-fourths), the onset of epilepsy occurs in the pediatric age group.^2^ Pediatric patients with epilepsy exhibit comorbidities that affect developmental progress and emotional health, including attention-deficit, learning disabilities, depression, and anxiety.^3^ At present, antiepileptic drugs (AEDs) are the main treatment for most patients with epilepsy.^4^ Although seizures can be partially controlled by most traditional AEDs, seizure control remains poor in many children. More than 30% of pediatric patients with epilepsy are not responsive to conventional AEDs and gradually develop refractory epilepsy.^5^, ^6^ Therefore, it needs new drug treatment options for people with epilepsy.

Lacosamide (LCM), approved in China in 2018, is a new type of AED that can selectively enhance the slow deactivation of voltage-gated sodium channels, block sustained sodium currents, suppress long-term high-frequency discharges during epileptic discharge, and has little effect on short-term high-frequency discharges. Therefore, it can stabilize the overexcited neuronal cell membrane and control epileptic discharge without affecting normal physiological function.^7^, ^8^

A systematic review revealed that LCM has good effectiveness and tolerability in various types of epilepsy in adults and children.^9^ A Brazilian study including refractory epilepsy showed a 73.9% reduction in seizure frequency of >50% after nine months of LCM treatment.^10^ Results of an LCM treatment study for children with focal epilepsy in China showed complete seizure control of 71.7% after 12 months of treatment.^11^ However, there is still insufficient research on the effectiveness and tolerability of LCM in Chinese children with focal or generalized epilepsy.

The pharmacokinetic variability of LCM was large.^12^-^14^ Various factors can affect the concentration of LCM in plasma, such as daily dose, age, weight, and sex.^15^ In addition, because LCM is mainly metabolized by CYP2C9, CYP2C19 and CYP3A4 enzymes in liver CYP450,^8^ a pharmacokinetics study of LCM in epilepsy patients shows that when used in combination with strong enzyme inducers, LCM has a linear dose concentration relationship, and serum concentration decreases.^13^, ^14^ The large variability in LCM and narrow therapeutic window necessitate therapeutic drug monitoring (TDM), thus ensuring optimal effectiveness and avoiding adverse effect, especially in pediatric patients. Of note, defining a specific reference range of LCM is meaningful. According to the Consensus Guidelines, the effective LCM therapeutic reference range for epilepsy is 1-10 mg/L.^16^ However, other ranges have been recommended, such as 2.0-7.0 µg/mL,^15^ 2.5-10 µg/mL,^17^ and 3-10 µg/mL,^18^ but the optimal therapeutic range remains undetermined. Therefore, attention should be given to the effect on plasma concentrations of LCM and more data about reference range is needed.

The aim of this study is to retrospectively analyze the effectiveness and tolerability of LCM as a monotherapy or adjunctive therapy for epilepsy in pediatric patients, provide more data about the reference range of LCM in the plasma, and identify the potential factors influencing the plasma concentrations of LCM.

## Methods

### Study design and ethics

This study was an single center retrospective trial. The Ethics Committee of the Second Affiliated Hospital of Zhejiang University School of Medicine approved the study (Program No: 20230374). Informed consent was waived due to the retrospective nature of the study, which was approved by the Ethics Committee and complied with regional regulatory requirements.

### Patient inclusion

This study retrospectively included patients who is admitted to the Second Affiliated Hospital of Zhejiang University School of Medicine from January to September, 2022. The inclusion criteria were as follows: (1) diagnosed with epilepsy; (2) receiving LCM monotherapy or adjunctive therapy; (3) patient who received routine TDM for LCM; and (4) aged <18 years old. For routine TDM practice in our hospital, blood samples were collected when the concentration of LCM was in a steady state, and bioanalysis was performed on an HPLC‒MS/MS system.^19^ We calculated the concentration-to-dose (CD) ratio (µg/mL per mg/kg) to adjust for body weight.

### Data collection

We collected patients with visit data at baseline, 3, 6, and 12 months, including age, sex, weight, seizure types, dosage regimen of LCM, TDM results, combined AEDs, treatment response, reported ADRs and reasons for treatment interruption. Effectiveness was assessed based on cumulative changes in seizure frequency at 3, 6, and 12 months. Adverse events (AEs) will be recorded based on the observations of parents or doctors.

### Definitions of clinical response

Baseline seizure frequency was calculated within 6 months before starting LCM treatment. According to the fourth-level effectiveness evaluation criteria established by the first National Epilepsy Academic Conference of the Chinese Medical Association,^20^ it is divided into the following: ① Complete control: No further seizures of any kind have occurred after taking the drug for more than twice the longest intermittent period before the drug was given. ② Basic control: the number of seizures has been reduced by more than 75% and symptoms have been alleviated. ③ Effective: the frequency of seizures have been reduced by 50% to 75% and the severity of seizures has also been reduced. ④ Ineffective: No significant difference compared to before treatment. Responders are those who have more than 50% fewer seizures than the baseline period, and patients with less than 50% reduction in the number of seizures compared with the baseline period are non-responders.

In addition, based on the research methodology of previous studies, this study also determined new reference ranges based on the range in which the majority of patients were located and the proportion of responders within this range.^15^, ^17^

### Statistical analysis

All data were statistically analyzed using SPSS software (version 26.0, IBM). The continuous results are presented as the mean and standard deviation, and the categorical results are presented as numbers and percentages. Pearson's chi-square test or Fisher's exact test were used to test the difference of categorical variables. The student's t test was used for continuous outcomes with normal distribution. The Mann‒Whitney U test was used to compare continuous variables without normal distribution. A P value of <0.05 was considered statistically significant.

## Results

### Patient characteristics

**Figure 1:**
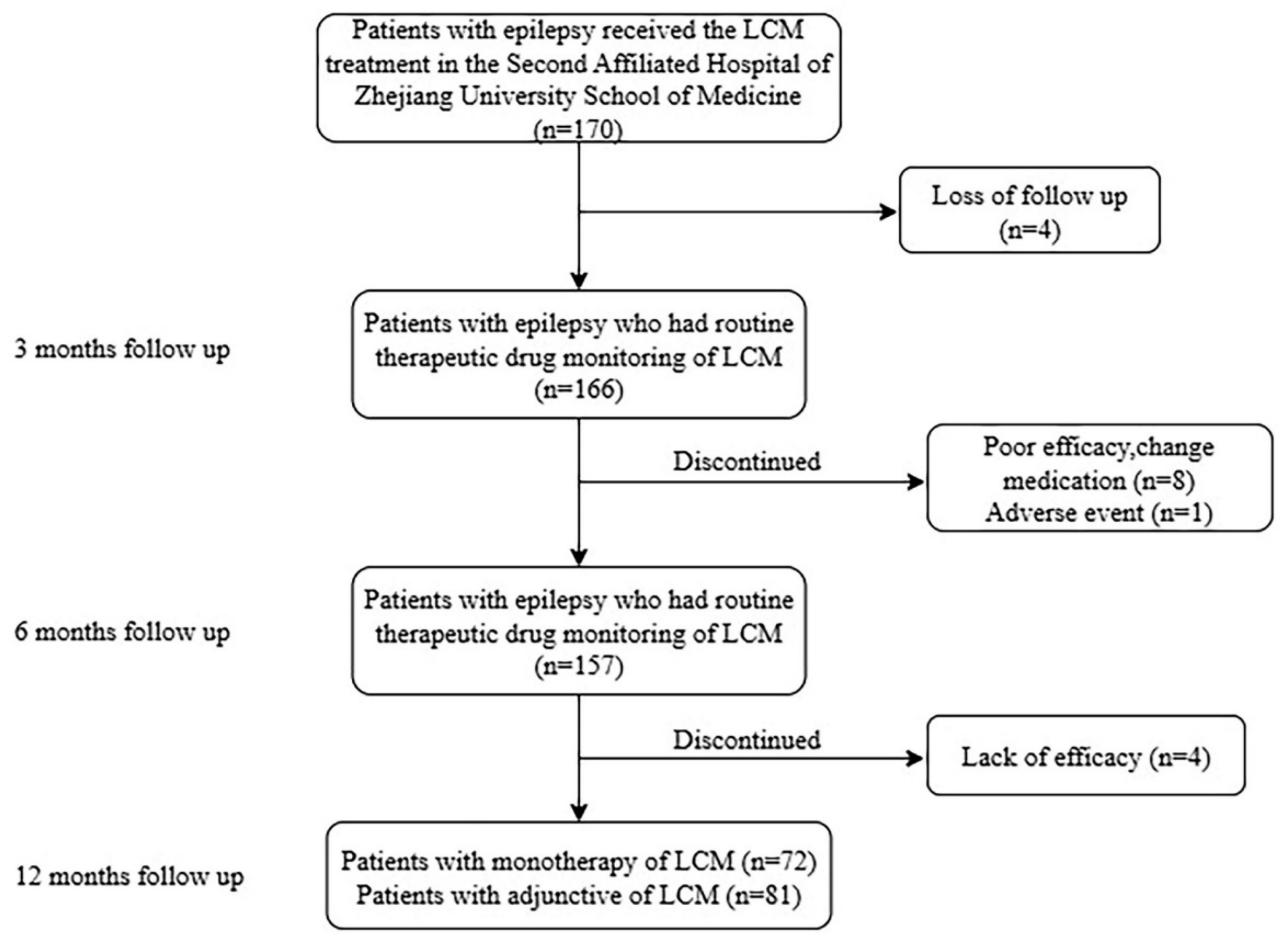
Numbers of patients who were eligible for the study.

A total of 334 concentrations obtained from 153 pediatric patients (58 females and 95 males) with epilepsy were included in the final analysis according to the inclusion and exclusion criteria (Figure 1). The characteristics of the patients are summarized in Table 1. Most patients were noted to have focal or generalized epilepsy. LCM was used in monotherapy in 72 patients, while the other patients used 1-4 concomitant AEDs.

### Effectiveness

The response rate of all patients at 12 months was 71.2%. The effect is good during the initial medication period, but later seizures occur or increase in frequency. Different age groups, types of epilepsy, genetic mutation issues, etc. show different effectiveness. The specific therapeutic effects of LCM are shown in Table 2.

### Tolerability

A total of 53 patients (34.6%) reported 70 AEs, most of which were mild and moderate. The main AEs reported were dizziness (n=17) and somnolence (n=10), irritability (n=8), distractibility (n=6), rash (n=5), headache (n=3), abdominal pain (n=2), undesirable weight gain (n=2), memory decline (n=2), poor appetite (n=2). Dizziness often occurs in the early stages of medication therapy or after increasing the dosage, and most ADRs are mild.

### TDM of LCM

**Figure 2:**
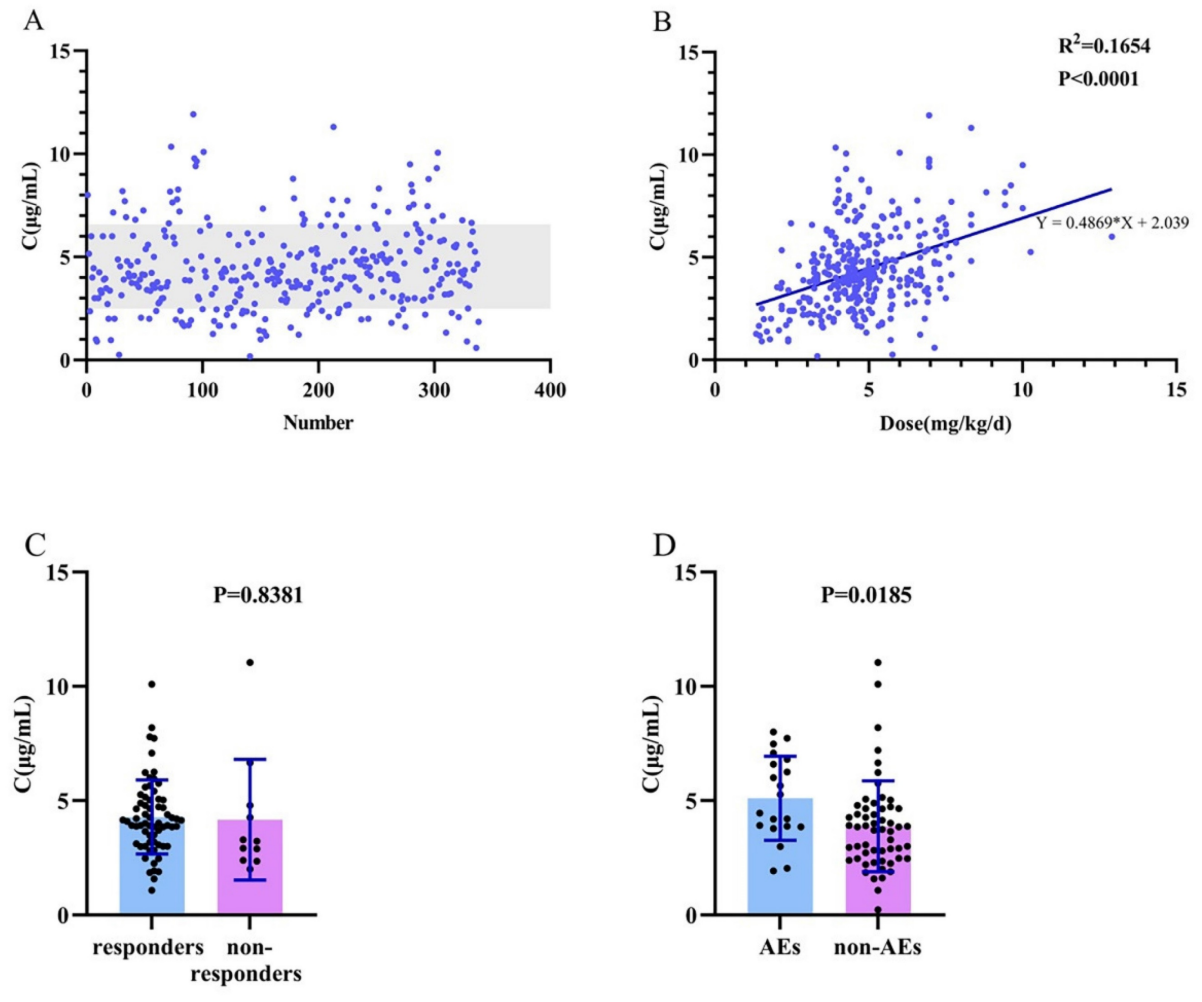
Plasma LCM C_0_ (µg/mL) measures of the maintenance dose in children with epilepsy. (A) C_0_ values in 153 children with epilepsy. (B) The correlation between C_0_ (µg/mL) and dose (mg/kg/d). (C) Comparison of C_0_ between responders and nonresponders to monotherapy. (D) Comparison of C_0_ between patients with adverse effects (AEs) and non- AEs in monotherapy.

C_0_ values found to be between 0.26 and 11.92 µg/mL (Figure 2A). Notably, approximately 77.1% of the monitored C_0_ values ranged from 2.5 to 6.5 µg/mL. Moreover, in the range of 2.5-6.5 µg/mL, 83.1% of patients showed a reduction of more than 50% in seizure frequency after 12 months of follow-up.

### Relationship between plasma concentrations and clinical outcomes

There was a weak but positive relationship between the plasma LCM C_0_ values and administration doses in both monotherapy and adjunctive therapy (R^2^=0.1654; P< 0.001; Figure 2B).

In patients treated with monotherapy, no significant difference was found in LCM C_0_ values between responders and non-responders (P=0.838; Figure 2C). Twenty patients had AEs, with a median LCM C_0_ value of 4.87 µg/mL, which was similar to the value of 3.80 µg/mL observed in without AEs (P=0.019; Figure 2D).

### Age, weight, sex, concomitant drugs, and the C_0_/dose ratio of LCM

**Figure 3:**
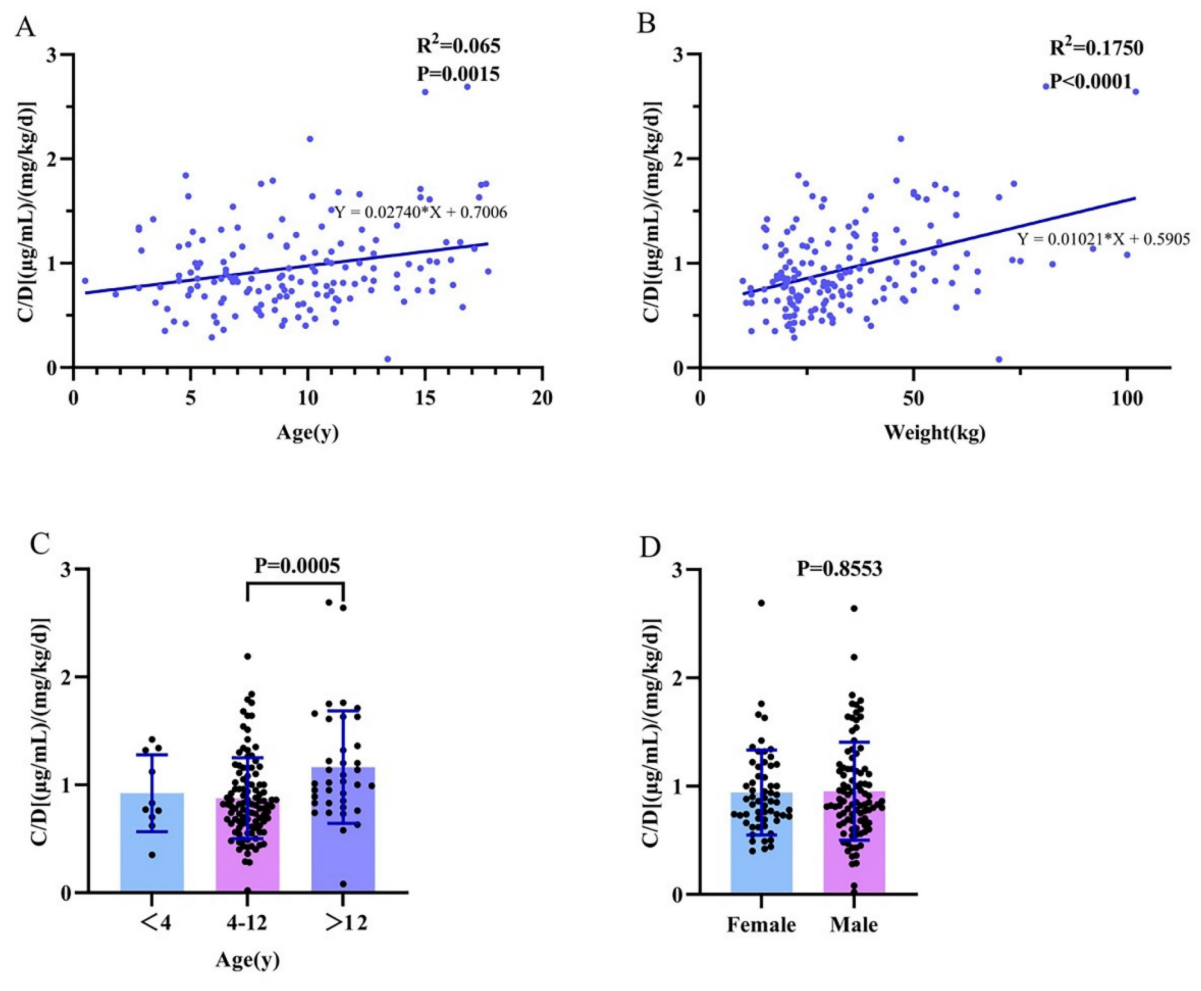
Association between C_0_/dose ratio [(µg/mL)/(mg/kg/d)] and various influencing factors. (A, C) Age; (B) Weight; (D) Sex.

In patients with LCM monotherapy or LCM adjunctive therapy, we found no correlation between age and the C_0_/dose ratio (R^2^=0.065; P=0.002; Figure 3A). There was a significant difference between the 4-12-year-old group and the group over 12 years old (P=0.001; Figure 3C). There was no significant difference between females and males (P=0.855; Figure 3D). When using multiple linear regression, it was found that age (P=0.017), weight (P<0.001), and dose (P=0.006) had statistically significant effects on C_0_/dose.

**Figure 4:**
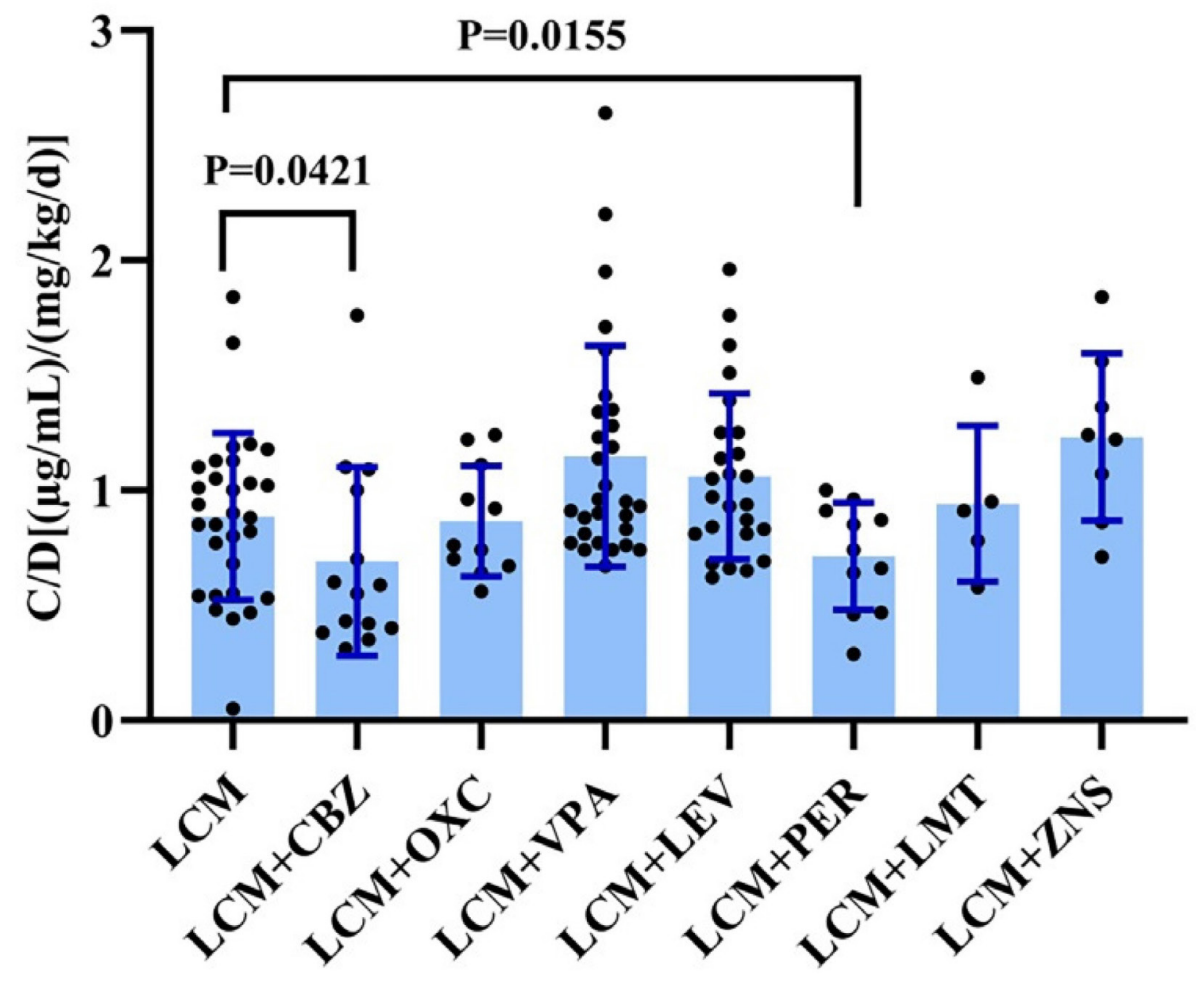
The C_0_/dose ratio of LCM in monotherapy and adjunctive therapy. CBZ: Carbamazepine; OXC: Oxcarbazepine; VPA: Sodium Valproate; LEV: Levetiracetam; PER: Perampanel; TPM: Topiramate; ZNS: Zonisamide.

We evaluated the impact of various combination therapies on the C_0_/dose of LCM compared to monotherapy (Figure 4). Notably, carbamazepine significantly decreased the C_0_/dose ratio of LCM (P=0.042; Figure 4). In this study, it is worth noting that perampanel also significantly decreased the C_0_/dose ratio of LCM (P=0.016; Figure 4). Interestingly, coadministration with other AEDs did not impact the C_0_/dose ratio of LCM (i.e., LCM + AEDs vs. LCM).

## Discussion

This retrospective study assessed the effectiveness and tolerability of LCM as monotherapy and adjunctive therapy in Chinese pediatric patients. Particularly, we optimized, for the first time, the TDM reference range of LCM for those children and evaluated how the demographic and clinical variables influence the plasma LCM concentrations.

In terms of effectiveness, Driessen JT *et al.*^21^ found that in 79 Dutch pediatric epilepsy patients, the effective rates of LCM were 60.5%, 67.9%, and 71.4% after 3, 12, and 24 months of follow-up, respectively. As the treatment time increases, the treatment effectiveness improves. Persistent adherence to long-term medication therapy is beneficial for reducing seizure frequency. Farkas V *et al.*^22^ found that the frequency of focal seizures per 28 days of LCM was reduced by 31.72% during maintenance and 30.18% during treatment compared with placebo. Sanmart í-Vilaplana F *et al.*^23^ found that 44.4% of children with epilepsy <18 years old in Spain had a reduced frequency of seizures by more than 50% after using LCM. Torleiv Svendsen *et al.*^17^ from Norway evaluated the efficacy in 227 patients, 29% of whom had a seizure frequency reduced by more than 50%. The results of these three studies show that the efficacy of LCM is poor, with an effective rate of less than 50%. In a study of LCM in China, Zhao T *et al.*^24^ found that 361 pediatric patients (72.2%) were effectively treated with lacosamide, and the seizure-free rate was 54.8%. Li Y *et al.*^15^ found that six months of additional LCM treatment reduced the frequency of seizures in 70% of patients by more than 50%, and the one-year treatment result was 81%. In China, LCM treatment reduced seizure frequency by ≥50% in more than 70% of patients. In this study, after 12 months of follow-up, 71.2% of patients achieved a reduction of more than 50% in seizure frequency, and 40.6% of patients were completely seizure-free. Interestingly, we found that the effectiveness in Chinese patients was higher than that in other countries, which is worth further research.

In terms of tolerability, our study found that the most common ADRs of LCM are dizziness and somnolence, which is similar to the studies by Farkas V^22^ and William Rosenfeld *et al.*^25^ However, Ben Menachem E *et al.*^26^ reported a significant number of nonneurological ADRs, such as nasopharyngeal inflammation and back pain, which were not reported in this study. It is worth noting that this study reported 8 cases of irritability and 6 cases of distractibility, which is not common. In addition, there was a significant difference in the concentration between the groups with and without AEs. The group with AEs had a higher plasma drug concentration. Therefore, it is necessary to monitor the plasma drug concentration and control it within an appropriate range to ensure effectiveness while reducing the occurrence of ADRs.

Another important result of this study was the optimization of the reference range for LCM plasma drug concentration. Torleiv Svendsen *et al.*^17^ from Norway found that the serum concentration in almost all patients showing a good treatment response was in the range of 2.5-10 µg/mL. This finding suggests that LCM is likely to be most clinically effective within this range. Therefore, they suggest using it as a reference range. A similar reference range of 2.25-8.75 µg/mL is used in Denmark.^27^ Yue Li *et al.*^15^ found that 92.1% of C_0_ values ranged from 2.0 to 7.0 µg/mL. Within this range, 71.4% of patients had no seizures. During LCM monotherapy, 36 measurements were recorded, and approximately 88.8% of C_0_ values ranged from 2.0 to 7.0 µg/mL. A total of 96.9% of patients had no seizures. Therefore, they suggest that C_0_ (2.0-7.0 µg/mL) may be feasible when LCM is used as monotherapy or adjunctive therapy for pediatric patients with epilepsy in China. We used a similar method to determine the range of LCM plasma concentrations, and 77.1% of the monitored C_0_ values ranged from 2.5 to 6.5 µg/mL. Moreover, in the range of 2.5-6.5 µg/mL, 83.1% of patients showed a reduction of more than 50% in seizure frequency after 12 months of follow-up. Therefore, we suggested that C_0_ (2.5-6.5 µg/mL) might be an alternative and more suitable when LCM is used as monotherapy or adjunctive therapy for pediatric patients with epilepsy in China.

Age and gender have been identified as factors that affect LCM pharmacokinetics in previous study.^28^ However, there is no difference observed in C_0_/dose between males and females (Figure 3D). In our study, of note, the C_0_/ dose ratio of LCM increased with age and weight when used alone or in combination with other AEDs (Figure 3A; Figure 3B). Specifically, there was a significant difference in C_0_/D between the 4-12-year-old group and the >12-year-old group. This may be due to the high metabolism of younger children, as drugs are absorbed, metabolized and excreted more rapidly in the body. As a result, younger children may require larger doses to achieve similar levels for the same body weight. Compared with monotherapy, CBZ is a strong CYP3A4 enzyme inducer that can enhance the metabolism of LCM in the liver and reduce the plasma drug concentration of LCM. Notably, the present study found that PER also decreased C_0_/D, a result not seen in previous articles, possibly due to the weak induction of PER on the CYP3A4 enzyme, one of the major metabolic enzymes of LCM. In addition, regression analysis revealed that the effects of dose, weight, CBZ, and PER on C_0_/D were significant.

This study still has several limitations. First, this was a single-center study, the external validity of its results may be limited. Second, 153 children were included, but they had variable therapy periods, and we had to rely on real-world clinical reporting rather than seizure frequency prospectively reported in patient diaries. Nevertheless, the real-world clinical findings in this study for effectiveness and tolerability, especially for LCM plasma monitoring in children, may be very useful for pediatric clinicians and TDM pharmacists when they try to tailor LCM dosages for precision therapy.

## Conclusion

This study found that LCM treatment used alone or with other AEDs in pediatric patients with epilepsy can reduce seizure frequency, with mild ADRs in some patients. We also identified several contributing factors to the variable C_0_/dose ratio of LCM in pediatric patients with epilepsy. Complex drug interactions between LCM and other concomitant AEDs were revealed. Of note, based on the data we analyzed, we proposed an alternative reference range of plasma LCM levels, that is, 2.5-6.5 µg/mL, for pediatric patients in China. Considering the existing study limitations, future research is needed.

## Figures and Tables

**Table 1:** Demographic and clinical characteristics of the patients

Characteristics	Value
N	153
Age(year)	
Median	9.1
IQR	5.3
Sex, n (%)	
M	95 (62.1%)
F	58 (37.9%)
Weight(kg)	
Median	30.0
IQR	22.5
Type of epilepsy, n (%)	
Focal	96 (62.7%)
Generalized	48 (31.4%)
Focal with generalized	5 (3.3%)
Unknown	4 (2.6%)
Dose (mg/kg/d)	
Median	4.55
IQR	1.6
Number of Concomitant AEDs, n (%)	
0	72 (47.1%)
1	58 (37.9%)
2	17 (11.1%)
3	4 (2.6%)
4	2 (1.3%)
Etiology of epilepsy*, n (%)	
Structural	66 (43.1%)
Genetic	19 (12.4%)
Infection	2 (1.3%)
Unknown	66 (43.1%)

IQR: Interquartile Range; M: male; F: female; * based on full etiological screening (e.g. imaging/genetic testing).

**Table 2:** Efficacy and tolerability of LCM

Variable	Number	Complete control	Basic control	Effective	Ineffective	P value	ADR	P value
**Age (y)**						0.691		0.572
<4	10 (6.5%)	3 (30.0%)	3 (30.0%)	0	4 (40.0%)		2 (20.0%)	
4-12	106 (69.3%)	48 (45.3%)	26 (24.5%)	10 (9.4%)	22 (20.8%)		37 (34.9%)	
>12	37 (24.2%)	18 (48.6%)	9 (24.3%)	4 (10.8%)	6 (16.2%)		14 (37.8%)	
**Sex**						0.038		0.750
Male	95 (62.1%)	50 (52.6%)	23 (24.2%)	5 (5.3%)	17 (17.9%)		32 (33.7%)	
Female	58 (37.9%)	19 (32.8%)	15 (25.9%)	9 (15.5%)	15 (25.9%)		21 (36.2%)	
**Type of epilepsy**						0.238		0.082
Focal	96 (62.7%)	47 (49.0%)	20 (20.8%)	8 (8.3%)	21 (21.9%)		33 (34.4%)	
Generalized	48 (31.4%)	15 (31.3%)	17 (35.4%)	6 (12.5%)	10 (20.8%)		16 (33.3%)	
Focal with generalized	5 (3.3%)	4 (80.0%)	0	0	1 (20.0%)		4 (80.0%)	
Unknown	4 (2.6%)	3 (75.0%)	1 (25.0%)	0	0		0	
**Therapeutic medication**						0.009		0.093
Monotherapy	72 (47.1%)	42 (58.3%)	14 (19.4%)	7 (9.7%)	9 (12.5%)		20 (27.8%)	
Adjunctive therapy	81 (52.9%)	27 (33.3%)	24 (29.6%)	7 (8.6%)	23 (28.4%)		33 (40.7%)	
**Gene**						0.328		0.829
Gene mutation	19 (12.4%)	5 (26.3%)	6 (31.6%)	3 (15.8%)	5 (26.3%)		7 (36.8%)	
No gene mutation	134 (87.6%)	64 (47.8%)	32 (23.9%)	11 (8.2%)	27 (20.1%)		46 (34.3%)	
**Time**						0.141		
3-months	166 (170)	95 (55.9%)	23 (13.5%)	9 (5.3%)	39 (22.9%)			
6-months	157 (170)	79 (46.5%)	35 (20.6%)	10 (5.9%)	33 (19.4%)			
12-months	153 (170)	69 (40.6%)	38 (22.4%)	14 (8.2%)	32 (18.8%)			
